# Brain functional connectivity alterations associated with neuropsychological performance 6–9 months following SARS‐CoV‐2 infection

**DOI:** 10.1002/hbm.26163

**Published:** 2022-12-02

**Authors:** Philippe Voruz, Alexandre Cionca, Isabele Jacot de Alcântara, Anthony Nuber‐Champier, Gilles Allali, Lamyae Benzakour, Patrice H. Lalive, Karl O. Lövblad, Olivia Braillard, Mayssam Nehme, Matteo Coen, Jacques Serratrice, Jean‐Luc Reny, Jérôme Pugin, Idris Guessous, Radek Ptak, Basile N. Landis, Dan Adler, Alessandra Griffa, Dimitri Van De Ville, Frédéric Assal, Julie A. Péron

**Affiliations:** ^1^ Clinical and Experimental Neuropsychology Laboratory, Faculty of Psychology University of Geneva Geneva Switzerland; ^2^ Department of Clinical Neurosciences, Neurology Department Geneva University Hospitals Geneva Switzerland; ^3^ Faculty of Medicine University of Geneva Geneva Switzerland; ^4^ Leenaards Memory Center Lausanne University Hospital and University of Lausanne Lausanne Switzerland; ^5^ Psychiatry Department Geneva University Hospitals Geneva Switzerland; ^6^ Diagnostic and Interventional Neuroradiology Department Geneva University Hospitals Geneva Switzerland; ^7^ Division and Department of Primary Care Medicine Geneva University Hospitals Geneva Switzerland; ^8^ Division of General Internal Medicine, Department of Medicine Geneva University Hospitals and Geneva University Geneva Switzerland; ^9^ Intensive Care Department Geneva University Hospitals Geneva Switzerland; ^10^ Neurorehabilitation Department Geneva University Hospitals Geneva Switzerland; ^11^ Rhinology‐Olfactology Unit, Otorhinolaryngology Department Geneva University Hospitals Geneva Switzerland; ^12^ Division of Pulmonary Diseases Geneva University Hospitals Geneva Switzerland; ^13^ Institute of Bioengineering, Center for Neuroprosthetics, Ecole Polytechnique Fédérale de Lausanne (EPFL) Lausanne Switzerland

**Keywords:** cognition, COVID‐19, functional connectivity, MRI, neuropsychological deficits

## Abstract

Neuropsychological deficits and brain damage following SARS‐CoV‐2 infection are not well understood. Then, 116 patients, with either severe, moderate, or mild disease in the acute phase underwent neuropsychological and olfactory tests, as well as completed psychiatric and respiratory questionnaires at 223 ± 42 days postinfection. Additionally, a subgroup of 50 patients underwent functional magnetic resonance imaging. Patients in the severe group displayed poorer verbal episodic memory performances, and moderate patients had reduced mental flexibility. Neuroimaging revealed patterns of hypofunctional and hyperfunctional connectivities in severe patients, while only hyperconnectivity patterns were observed for moderate. The default mode, somatosensory, dorsal attention, subcortical, and cerebellar networks were implicated. Partial least squares correlations analysis confirmed specific association between memory, executive functions performances and brain functional connectivity. The severity of the infection in the acute phase is a predictor of neuropsychological performance 6–9 months following SARS‐CoV‐2 infection. SARS‐CoV‐2 infection causes long‐term memory and executive dysfunctions, related to large‐scale functional brain connectivity alterations.

## Graphical abstract

One hundred and ten patients, with either severe, moderate, or mild disease in the acute phase underwent neuropsychological and olfactory tests, as well as completed psychiatric and respiratory questionnaires at 223 ± 42 days postinfection. Additionally, a subgroup of 50 patients underwent functional magnetic resonance imaging. The severity of the infection in the acute phase is a predictor of neuropsychological performance 6–9 months following SARS‐CoV‐2 infection. SARS‐CoV‐2 infection causes long‐term memory and executive dysfunctions, related to large‐scale functional brain connectivity alterations.

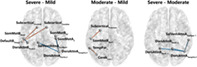

## 1 INTRODUCTION

The World Health Organization recently defined the long‐term consequences of SARS‐CoV‐2 infection as *post‐COVID‐19 condition*. This refers to a multisystem condition that occurs in individuals with a history of probable or confirmed SARS‐CoV‐2 infection, usually 3 months after onset of COVID‐19, with symptoms that last for at least 2 months and cannot be explained by an alternative diagnosis. To date, at least 52 clinical or biological signs have been listed (Tran et al., 2021), impacting eight different systems: pulmonary, cardiovascular, hematological, renal, endocrine, gastrointestinal, dermatological, and neuropsychiatric (Nalbandian et al., 2021).

This constellation of symptoms persists well after the acute phase of the infection and includes cognitive disorders (for a review, see Vanderlind et al., 2021). Observations suggest impairment of various cognitive functions up to 3 months following COVID‐19, with disruption of global cognitive efficiency (Alemanno et al., 2021; Amalakanti et al., 2021; Beaud et al., 2021; Blazhenets et al., 2021; De Lorenzo et al., 2020; Ferrucci et al., 2021; Kas et al., 2021; Negrini et al., 2021; Ortelli et al., 2021; Pirker‐Kees et al., 2021; Pistarini et al., 2021; Raman et al., 2021; Solaro et al., 2021; Udina et al., 2021), memory functions (Almeria et al., 2020; Hampshire et al., 2021; Jaywant et al., 2021; Whiteside et al., 2021; Woo et al., 2020), attention (Alemanno et al., 2021; Almeria et al., 2020; Hampshire et al., 2021), executive functions (Alemanno et al., 2021; Tay et al., 2021; Whiteside et al., 2021; Woo et al., 2020), logical reasoning (Hampshire et al., 2021), and language (Alemanno et al., 2021; Almeria et al., 2020; Whiteside et al., 2021). The etiopathogenesis of these disorders remains subject to debate, but three hypotheses have already been postulated. To date, the most plausible according to the literature seems to be an indirect/mediated damage may result from an excessive immune or inflammatory reaction. This is supported by evidence of hyperinflammation with features of cytokine storm syndrome (Cron et al., 2021), and by studies showing a link between neuropsychiatric symptoms and immune data (Mazza et al., 2020), as well as recent evidences from wide histopathological cohorts, suggesting an extensive glia activation and infiltration of CD4/8pos lymphocytes within the perivascular spaces (Matschke et al., 2020; Schwabenland et al., 2021; Thakur et al., 2021). That said, all three hypotheses can be supported by positron emission tomography (PET) studies revealing patterns of hypometabolism in the olfactory, frontal and limbic systems (Delorme et al., 2020; Guedj et al., 2021; Hosp et al., 2021). There is also the potential impact of the post‐resuscitation / intensive care unit (ICU) syndrome in patients whose symptoms were sufficiently severe to require such treatment. Cognitive deficits after ICU, associated with mechanical ventilation, have been demonstrated in other pathologies and are increasingly recognized (Jackson et al., 2007; Kohler et al., 2019) (for a recent review, see Sakusic et al., 2018). Interestingly, this review (Sakusic et al., 2018) found that the factors that predicted impaired cognition and structural brain damage after hospitalization in ICU were delirium and its duration. Based on these reviews, medication (sedatives and analgesics), mechanical ventilation, extracorporeal membrane oxygenation, trophic feeding, intraoperative hypotension, and hypoxia appear not to influence the likelihood of long‐term cognitive impairment (Sakusic et al., 2018).

The impact of respiratory severity in the acute phase of COVID‐19 on chronic neuropsychological symptoms has yet to be clarified/determined. Nevertheless, some studies using validated neuropsychological testing approaches to explore the consequences of SARS‐CoV‐2 infection have shed some light on this issue. For example, Woo et al. (2020) and Almeria et al. (2020) compared patients who benefited from oxygen therapy with those who did not. Woo et al. (2020) found no differences, whereas Almeria et al. (2020) reported significant differences on verbal memory, visual memory, working memory, processing speed, executive function, and global cognition. Reduced performances for executive functions were only observed in ICU patients. Alemanno et al. (2021) observed better cognitive scores among patients who had been under sedation and ventilated in the ICU, compared to patients who had been hospitalized without oxygen therapy. Nevertheless, the presence of methodological limitations reduces the extent to which inferences can be drawn about the potential impact of respiratory severity in the acute phase on chronic neuropsychological deficits. Moreover, only a small number of studies have simultaneously assessed chronic neuropsychological symptoms and carried out neuroimaging. In particular, to date, few studies have investigated functional connectivity in patients in long‐term following SARS‐CoV‐2 infection (>3 months postinfection), or only in the acute phase (Benedetti et al., 2021; Esposito et al., 2022; Fischer et al., 2022; Yildirim et al., 2022) and considering psychiatric (Benedetti et al., 2021) or olfactory symptoms (Esposito et al., 2022; Yildirim et al., 2022). Nevertheless, Fu et al. (2021) identified pattern of functional connectivity, associated with post‐traumatic stress disorder symptoms, revealing modifications in the sensorimotor and visual networks. Zhang et al. (2022) focused on analysis of intraconnectivity and interconnectivity of the default mode network (DMN) and revealed a higher interconnectivity of the DMN in patients reporting long‐term symptoms following SARS‐CoV‐2 infection. To date, no study has assessed brain functional connectivity in relation with neuropsychological performances as function of the severity of the acute infection.

In this context, the objective of the present study was to test whether differences in neuropsychological performances at 6–9 months postinfection were associated with modifications in functional brain networks, considering the severity of the respiratory symptoms in the acute phase. To this end, patients without clinical history that could induce neuropsychological deficits prior to infection with SARS‐CoV‐2 underwent a comprehensive assessment that probed multiple cognitive domains, emotion recognition, psychiatric symptoms, dyspnea, and olfaction. They were divided into three groups according to the respiratory severity of the disease in the acute phase: severe (ICU hospitalization; *n* = 24), moderate (conventional hospitalization; *n* = 42), and mild (no hospitalization; *n* = 44). Of these patients, 50 agreed to undergo MRI, for which structural visual and functional connectivity analyses were performed.

In view of our objectives, we developed two hypotheses. First, we expected differences in neuropsychological performances and modifications of the cerebral functional connectivity to be a function of disease severity in the acute phase (Hampshire et al., 2020), although moderate and mild patients might also exhibit deficits (Alemanno et al., 2021; Woo et al., 2020). Second, based on previous observation of altered connectivity patterns in the long‐term following SARS‐CoV‐2 infection (Fu et al., 2021; Zhang et al., 2022), we suspected that relationships between neuropsychological scores and changes in functional brain connectivity could be observed as a function of severity.

## 2 METHODS

### 2.1 Participants

Patients were selected among all the patients from the Geneva University Hospitals (HUG) that showed evidence of a SARS‐CoV‐2 infection (between March 2020 and May 2021) either by positive polymerase chain reaction (PCR) from nasopharyngeal swab and/or by positive serology while being included according to the exclusion criteria (see below). Patients were divided into 3 groups and included to the study at 223.07 ± 41.69 days postinfection: 24 patients who had been admitted to ICU during the acute phase of the infection (severe), 42 patients who had been hospitalized but did not require mechanical ventilation (moderate), and 44 patients who had tested positive but had not been hospitalized (mild). Of these patients, 50 agreed to undergo MRI scans (severe: *n* = 9, moderate: *n* = 21, mild: *n* = 20) (see Table 1).

**TABLE 1:** Sociodemographic data and medical history

	Mild	Moderate	Severe	*p*‐Value″
	*n* = 44	*n* = 42	*n* = 24	
Mean age in years (±*SD*)	56.57 (±7.23)	56.50 (±9.58)	62.08 (±12.03)	.078
Mean education level [1–3] (±*SD*)	2.72 (±0.45)	2.64 (±0.58)	2.50 (±0.59)	.373
Gender (% women)	34.10	35.70	20.80	.420
Handedness (% right handed)	97.70	92.90	95.80	.553
Mean days of hospitalization (±*SD*)	‐	12.00 (±12.87)	40.13 (±32.07)	‐
Diabetes in %	2.30	9.50	20.80	.083
Smoking in %	11.40	2.40	4.20	.206
History of respiratory disorders in %	11.40	11.90	25.00	.259
History of cardiovascular disorders in %	13.60	14.30	25.00	.432
History of neurological disorders in %	0	0	0	1
History of psychiatric disorders in %	2.30^a^	2.40^a^	4.20^a^	.887
History of cancer in %	0	0	0	1
History of severe immunosuppression in %	0	0	0	1
History of developmental disorders in %	0	0	0	1
Chronic kidney disease in %	0	0	8.3	.026*
Sleep apnea syndrome in %	9.10	11.90	29.20	.067

*Note*: *ns*: not significant; *SD*: standard deviation. “Statistical analysis performed: Kruskal–Wallis or chi^2^.”

^a^ Treated depression more than 10 years prior to COVID‐19.

* Significant between subgroups following a Chi^2^ analysis.

The required number of participants in each group was determined by a power analysis involving the comparison of two means. This analysis was based on the literature evaluating the short‐term neuropsychological effects of COVID‐19 in mild patients (Woo et al., 2020). To achieve the desired statistical power (1 − *β*) of 90% and risk of Type I error (*α*) of 0.05, results indicated that for a one‐sided hypothesis, 13 participants would be needed in each group and for a bilateral hypothesis 18. As we planned to perform nonparametric analyses, we had to increase the sample size by 15% (Lehmann, 2012), resulting in a minimum of 15 participants per group in the case of one‐sided hypothesis and 21 participants per group in the case of bilateral hypothesis.

**FIGURE 1:**
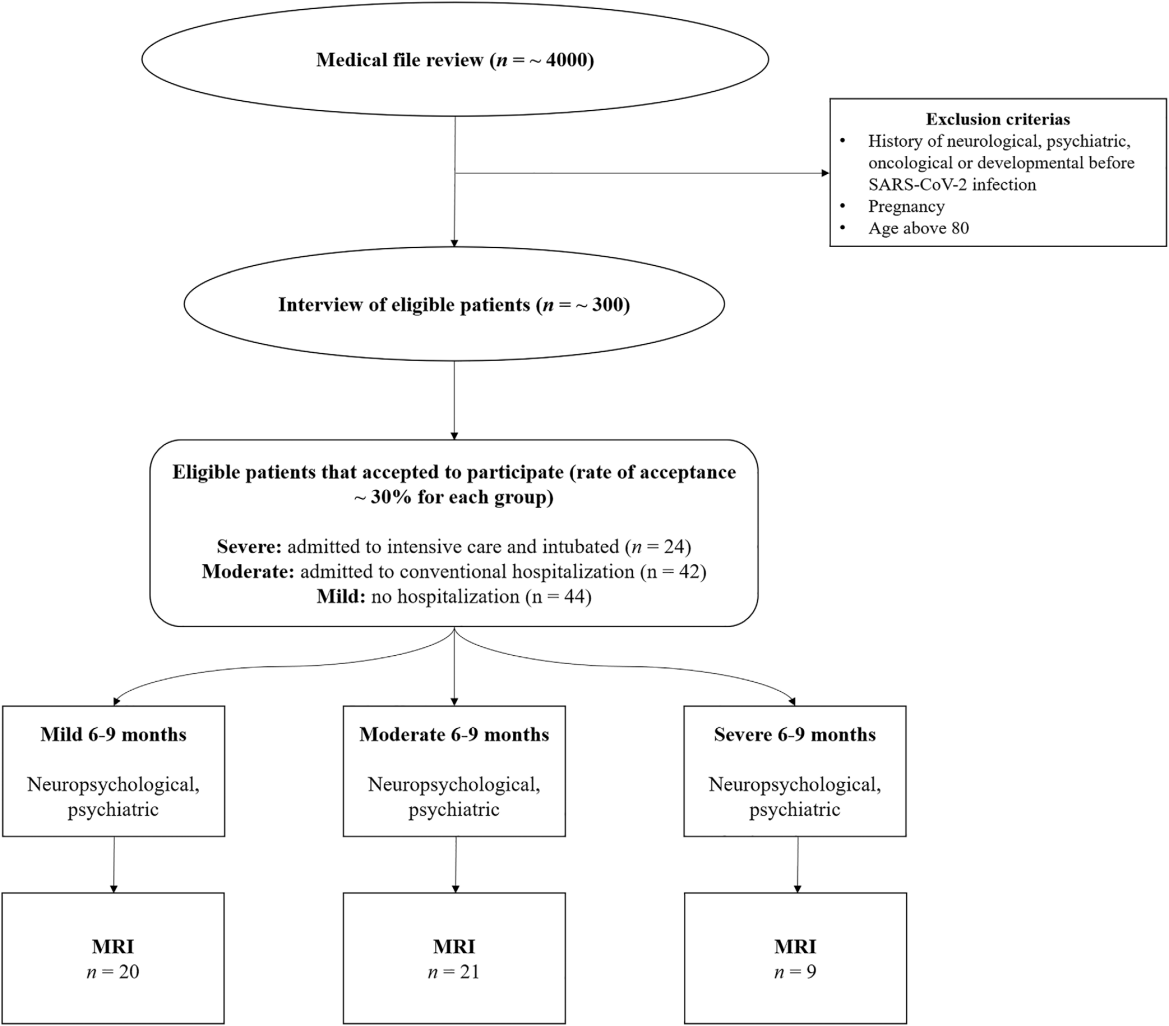
Flowchart of the study

The mild and moderate groups were matched during the screening‐inclusion process to the severe group for median age (mild = 57.50 years; moderate = 56.50 years; severe = 60 years), sociocultural level, and clinical variables (except for chronic renal failure) due to a limited number of available patients who were in ICU and met our exclusion criteria. Participants (*n* = 50) who underwent MRI were not matched during the screening‐inclusion process, and all patients that agreed for the MRI study were included. Nevertheless, the groups were still comparable on sociodemographic characteristics (except gender) and severity. Participants were recruited via CoviCare program (Nehme et al., 2021) following patients with post‐COVID symptoms in Geneva, Switzerland (MN, OB, and IG), as well as from registers from another study (LB). For each patient, we carried out a medical file review, followed by a telephone call inviting the patient to take part in the study, if all the eligibility criteria were met. Exclusion criteria were a history of neurological issues, psychiatric disorders (two of the included participants had had an episode of depression more than 10 years before their SARS‐CoV‐2 infection), cancer (to exclude possible chemotherapy‐ and radiotherapy‐related cognitive impairment (Cascella et al., 2018)), neurodevelopmental pathologies, pregnancy, and age above 80 years (see Figure 1).

### 2.2 General procedure and ethics

A flowchart displaying the successive stages of the study according to the eligibility criteria for each experimental group is provided in Figure 1.

After being given a full description of the study, participants provided their written informed consent. The study was conducted in accordance with the Declaration of Helsinki, and the study protocol was approved by the cantonal ethics committee of Geneva (CER‐02186).

### 2.3 Neuropsychological assessment and other clinical outcomes

The experimental design and tests used are comparable to those used in a previous published study (Voruz, Allali, et al., 2022; Voruz, Cionca, et al., 2022; Voruz, de Alcântara, et al., 2022).

A comprehensive neuropsychological battery (based only on tests of norms validated in a French‐speaking population) was administered in French to participants 6–9 months after their positive PCR test result. This battery included a series of tests and questionnaires that assessed most of the domains of cognition, emotion recognition, fatigue, and quality of life (see paragraph below). The tests were administered by clinical psychologists (mean duration: approximately 180 min), and the questionnaires were administered online via Qualtrics software (Qualtrics, Provo, UT) (mean duration: approximately 60 min).

#### 2.3.1 Executive functions

The Stroop task, Trail Making Test, and categorical and lexical verbal fluency from the GREFEX battery (Roussel & Godefroy, 2008) were administered to evaluate inhibition, shifting, and updating, in accordance with Miyake et al. (2000). Verbal working memory and visuospatial working memory were assessed with the backward digit span (Drozdick et al., 2018) and backward Corsi tests (Kessels et al., 2000). We also administered computer‐based tasks designed to gauge focused attention, divided attention, phasic alertness, working memory, and incompatibility, using version 2.1 of the Test for Attentional Performance (Zimmermann & Fimm, 2002).

#### 2.3.2 Memory systems

Short‐term memory was assessed with forward digit spans (Drozdick et al., 2018) and the Corsi test (Kessels et al., 2000). Verbal episodic memory was assessed with the 16‐item Grober and Buschke free/cued recall (RL/RI 16) paradigm (Grober & Buschke, 1987), as it distinguishes between the cognitive subprocesses of encoding, storage, and recall (Van der Linden et al., 2004). Visual episodic memory was assessed with the delayed recall of the Rey–Osterrieth Complex Figure test (Meyers & Meyers, 1995).

#### 2.3.3 Instrumental functions

Language was assessed with the BECLA battery (Macoir et al., 2016), ideomotor praxis with a short validated battery (Mahieux‐Laurent et al., 2009), visuoconstructive abilities with the Rey–Osterrieth Complex Figure test (Meyers & Meyers, 1995), and visuoperceptual functions with four subtests from the Visual Object and Space Perception battery (Warrington & James, 1991) that measured object perception (fragmented letters, object decision) and spatial perception (localization of numbers, analysis of cubes).

#### 2.3.4 Logical reasoning

This was assessed using the Puzzle and Matrices subtests of the Wechsler Adult Intelligence Scale–Fourth Edition (Wechsler, 2008).

#### 2.3.5 Emotion

Multimodal emotion recognition was assessed with the Geneva Emotion Recognition Test (GERT) (Schlegel & Scherer, 2016). Participants watched 42 video clips in which 10 actors displayed 14 different emotions (pride, fun, happiness, pleasure, relief, interest, anger, irritation, fear, anxiety, disgust, despair, sadness, surprise) while expressing nonverbal content. After each clip, participants were asked to choose one emotion from the list of 14 that best described the emotion displayed by the actor.

#### 2.3.6 Anosognosia and cognitive complaints

We administered the Cognitive Complaints Questionnaire (QPC) (Thomas‐Antérion et al., 2004) and the Behavior Rating Inventory of Executive Function‐Adult Version (BRIEF‐5) (Roth et al., 2005). To quantify anosognosia, we calculated a self‐appraisal discrepancy (SAD) score for each memory and executive domain evaluated by the QPC and BRIEF‐5 (Leicht et al., 2010; Rosen et al., 2010; Tondelli et al., 2018). First, we calculated standardized scores for the cognitive complaints, dividing the raw scores of the self‐report questionnaires into four categories: 0 = normal behavior, 1 = limited influence on daily life, 2 = noticeable influence on daily life, and 3 = substantial influence on daily life. We then subtracted each of these standardized scores from the standardized score for the relevant function. For example, if a patient reported no memory disorders (QPC score = 3), but performed very poorly on the RL/RI 16 delayed free recall test (score = 0), he or she would be deemed to exhibit anosognosia for memory dysfunction: 0–3 = −3. SAD scores could therefore range from −3 to 3, and any score below 0 indicated anosognosia.

#### 2.3.7 Symptom validity

The BRIEF‐A was used to measure the validity of patients' responses, as well as the presence of any noncredible symptoms (Abeare et al., 2021; Harrison et al., 2021).

### 2.4 Other clinical outcomes

We collected patients' sociodemographic data and medical history. Psychiatric data (including current fatigue, insomnia, and somnolence), dyspnea, and data on olfactory abilities at the time of the interview were also collected. Finally, a neurological assessment of CNS and peripheral nervous system functions and walking was carried out by two certified neurologists (FA and GA).

#### 2.4.1 Sociodemographic and clinical data

In addition to age, collected during the inclusion interview, we recorded patients' gender, handedness, and education level. To complement information about previous neurological, psychiatric, and developmental conditions and cancer collected during the inclusion interview, we asked patients about previous cardiovascular disease, respiratory disorders, immunosuppression status, sleep apnea syndrome, diabetes, and smoking. Participants were asked to describe the symptoms they had experienced, both during the acute phase of the infection and currently (6–9 months postinfection), and the number of days they had spent in hospital, where relevant.

#### 2.4.2 Psychiatric data

Depressive symptoms were assessed with the Beck Depression Inventory‐Second edition (Beck et al., 1996), anxiety with the State–Trait Anxiety Inventory (Spielberg et al., 1993), apathy and its distinct subtypes with the Apathy Motivation Index (Ang et al., 2017), PTSD with the Posttraumatic Stress Disorder Checklist for DSM‐5 (Ashbaugh et al., 2016), manic symptoms with the Goldberg Mania Inventory (Goldberg, 1993), dissociative symptoms in the patient's daily life with the Dissociative Experience Scale (Carlson & Putnam, 1993, current stress perception with the Perceived Stress Scale – 14 items (Lesage et al., 2012), cognitive reappraisal of an emotional episode and expressive emotional suppression abilities with the Emotion Regulation Questionnaire (Gross & John, 2003), and susceptibility to others' emotions with the Emotional Contagion Scale (Doherty, 1997). Finally, fatigue was assessed with the French version of the Fatigue Impact Scale (Debouverie et al., 2007), potential sleeping disorders with the Insomnia Severity Index (Morin, 1993), and symptoms of sleepiness in daily life with the Epworth Sleepiness Scale (Johns, 1991).

#### 2.4.3 Dyspnea

Dyspnea was evaluated with a self‐report questionnaire (Beaumont et al., 2018) that distinguishes between the physical and affective aspects of self‐reported dyspnea.

#### 2.4.4 Olfaction

Olfactory performance was measured with the Sniffin' Sticks test battery. For each odor, patients had to choose between four descriptors in a multiple‐choice task. Participants' scores ranged from 0 to 16 (Kobal et al., 2000).

### 2.5 Symptom validity and presence of noncredible symptoms

First, to validate our neuropsychological measurements, we checked the validity of patients' symptoms. Both the measurement of symptom validity (i.e., congruence) and the measurement of noncredible symptoms with the BRIEF‐A showed good‐to‐excellent results for all participants, validating the results of the neuropsychological tests and the psychiatric symptom questionnaires.

### 2.6 Neuroimaging processing

#### 2.6.1 Image acquisition

A total of 50 participants (mild: *n* = 20; moderate: *n* = 21; severe: *n* = 9) underwent MRI scans at the CIBM Center for Biomedical Imaging in Geneva, on a Siemens Magnetom PrismaFit 3 tesla scanner. Analysis revealed no significant differences between the mild, moderate, and severe groups on age (mild: 55.18 ± 8.58, moderate: 54.94 ± 12.93, severe: 57.80 ± 12.49, *p* = .885), sociocultural level (mild: 2.76 ± 0.44, moderate: 2.78 ± 0.43, severe: 2.80 ± 0.42, *p* = .978) or handedness (one left‐handed in the mild group), whereas a significant difference was observed for gender (*p* = .049), with a higher proportion of men in the severe group as compared to mild and moderate. Intergroup analysis also failed to reveal any significant differences either on the interval between infection and MRI (mild: 254.18 ± 39.52 days; moderate: 287.17 ± 45.24 days; severe: 280.80 ± 54.06 days; *p* = .058) and the interval between neuropsychological testing and MRI (mild: 30.47 ± 20.66 days; moderate: 39.83 ± 26.23 days; severe: 51.39 ± 25.67 days; *p* = .112). Data from five patients were excluded due to high movement and/or poor registration. Structural images were obtained with a T1‐weighted (T1w) magnetization‐prepared rapid acquisition gradient echo sequence with an isotropic voxel size of 0.9375 × 0.9375 × 0.9 mm^3^ (SI 1). Resting‐state functional images were acquired through a multiband accelerated echoplanar sequence with an isotropic voxel size of 2.5 × 2.5 × 2.5 mm^3^, 64 slices, and repetition time of 1 s for a total of 7 min 59 s of acquisition time (480 volumes; SI 2). Additionally, susceptibility weighted images and fluid‐attenuated inversion recovery images were acquired for a visual investigation of brain structural damage (SI 1).

Preprocessing was performed using *fMRIPrep* 20.2.3 (Esteban et al., 2019), which is based on Nipype 1.6.1 (Gorgolewski et al., 2011).

#### 2.6.2 Anatomical preprocessing

Each T1w volume was corrected for intensity nonuniformity using N4BiasFieldCorrection v2.1.0 (Tustison et al., 2010), and skull‐stripped using antsBrainExtraction.sh v2.1.0 (using the OASIS template). Spatial normalization to the ICBM 152 Nonlinear Asymmetrical template version 2009c (Fonov et al., 2009) was performed through nonlinear registration with the antsRegistration tool of ANTs v2.1.0 (Avants et al., 2008), using brain‐extracted versions of both T1w volume and template. Brain tissue segmentation of cerebrospinal fluid, white matter (WM), and gray matter was performed on the brain‐extracted T1w using fast (Zhang et al., 2000) (FSL v5.0.9).

#### 2.6.3 Functional preprocessing

Functional data were slice‐time corrected using 3dTshift from AFNI v16.2.07 (Cox & Hyde, 1997), and motion corrected using mcflirt (FSL v5.0.9 (Jenkinson et al., 2002)). This was followed by FLIRT (FSL) coregistration to the corresponding T1w images using boundary‐based registration (Greve & Fischl, 2009) with six degrees of freedom. Motion‐correcting transformations, BOLD‐to‐T1w transformation and T1w‐to‐template (MNI) warp were concatenated and applied in a single step using antsApplyTransforms (ANTs v2.1.0), configured with Lanczos interpolation. Framewise displacement (Power et al., 2014) was calculated for each functional run using Nipype and volumes with a framewise displacement greater than 0.7 mm were excluded (SI 3).

Many internal operations of fMRIPrep use Nilearn (Abraham et al., 2014), principally within the BOLD‐processing workflow. For more details of the pipeline, see the section corresponding to workflows in the *fMRIPrep* documentation.

In addition, the preprocessed fMRI timeseries were detrended and the first five lowest frequency basis of the discrete cosine transform (from 0.001 to 0.005 Hz) were regressed from the signal. A low‐pass filter with cut‐off frequency at 0.15 Hz was applied and fMRI volumes were spatially smoothed with a Gaussian kernel and a full‐width‐at‐half‐maximum of 4 mm.

#### 2.6.4 Behavioral statistical analyses

We compared the three groups (severe, moderate, mild) on the raw data for each neuropsychological, psychiatric, olfactory, fatigue, and dyspnea variable. Given the nonparametric distribution of the samples (as measured with Shapiro–Wilks tests), we used nonparametric Kruskal–Wallis tests. For significant (*p* < .050) measures, Mann–Whitney *U* tests were performed for the 2 × 2 comparisons, with Benjamini–Hochberg false discovery rate (FDR) corrections as function of each domain (cognition, psychiatry) and each Mann–Whitney pairwise comparison (mild vs. severe; mild vs. moderate; moderate vs. severe).

#### 2.6.5 Neuroimaging statistical analysis

##### Structural MRI inspection

First, the neuroimaging data were visually analyzed to look for noticeable brain lesions such as microbleeds and WM damages. Groups were compared on the total number of microbleeds and impact on WM, with the Wahlund scale (Wahlund et al., 2001). Second, voxel‐based morphometry (VBM) analyses (Ashburner & Friston, 2000; Mechelli et al., 2005) were performed by computing the proportion of grey and WM voxels within the whole brain mask or within the fMRI parcellation (see below) and by comparing the outcome between the groups. Statistical differences were assessed with ANCOVA and considering age, gender and sociocultural level as covariates.

##### fMRI statistical analysis

The processed functional time courses were averaged into 156 regions of interest (100 cortical regions (Schaefer et al., 2018) that can be associated with 17 resting‐state networks (Yeo et al., 2011), 34 cerebellar regions (Diedrichsen et al., 2009) and 22 subcortical regions (Amunts et al., 2013)) to perform functional connectivity analyses considering the whole brain. Measures of functional connectivity were converted into *z* scores with the Fisher *z* transformation and compared using two‐sample *t* tests to investigate differences between groups. The normality of functional connectivity measures was confirmed with Shapiro–Wilk tests and *p* values were FDR corrected for multiple comparisons.

#### 2.6.6 Relationship between neuropsychological scores and brain connectivity

A partial least squares correlation (PLSC) approach was used to evaluate multivariate associations between neuroimaging and behavioral data (McIntosh & Lobaugh, 2004). This technique estimates latent components that consist out of linear combinations of brain functional connectivity and neuropsychological scores, respectively, to maximize their covariance across participants. The significance of the latent components was evaluated with permutation testing (1000 permutations), and the stability of the feature weights (called saliences) was assessed through bootstrapping (500 samples). Furthermore, we computed the imaging and behavioral loadings defined by the Pearson's correlation between the original neuropsychological and functional connectivity values, and their corresponding PLSC weights. Only the neuropsychological scores surviving the FDR correction in the intergroup comparison were considered in this analysis along with the whole brain functional connectivity. Three PLSC analyses were conducted. First, the data were observed in the whole group to identify general associations between behavioral and neuroimaging data. Then, a group‐PLS analysis was performed considering the group based on the severity. Finally, we repeated the analyses within each individual group to confirm the results from the group‐PLS approach. PLSC analyses were performed using the myPLS toolbox (https://github.com/danizoeller/myPLS).

## 3 RESULTS

### 3.1 Neuropsychological symptoms as a function of disease severity

**FIGURE 2:**
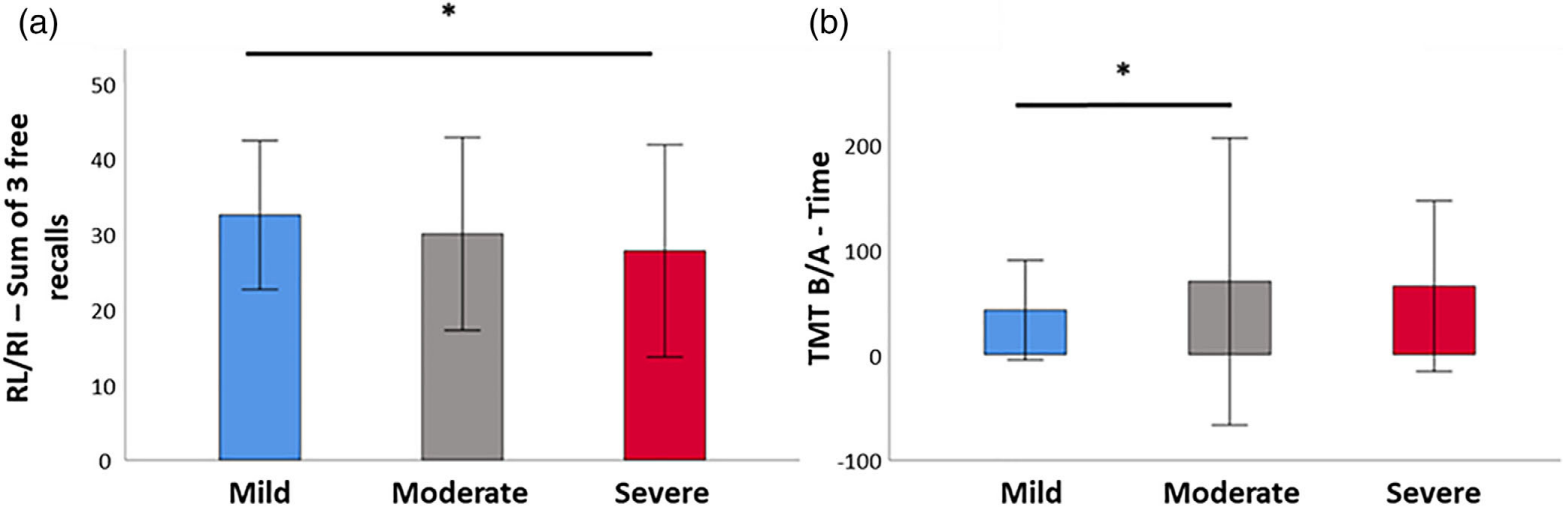
Intergroup comparisons for neuropsychological testing (after false discovery rate [FDR] correction). (a) Severe patients performed significantly more poorly than mild patients on the RL/RI 16—Sum of three free recalls. (b) Moderate patients had significantly higher interference scores than mild patients on the TMT B/A—Time

The three groups differed significantly on (i) memory encoding (RL/RI 16—immediate recall; *H* = 17.34, *p* < .001); (ii) long‐term episodic verbal memory (RL/RI 16—Sum of three free recalls; *H* = 9.39, *p* = .009; sum of three total recalls; *H* = 6.42, *p* = .040; delayed free recall; *H* = 11.10, *p* = .004); (iii) inhibition (Stroop Interference—Time; *H* = 7.61, *p* = .022); (iv) mental flexibility (TMT B—Time; *H* = 10.20, *p* = .006; TMT B—Perseverations; *H* = 13.07, *p* = .002; TMT B‐A—Time; *H* = 9.96, *p* = .007); (v) logical reasoning (WAIS IV—Puzzle; *H* = 6.72, *p* = .035; WAIS IV—Matrix; *H* = 6.47, *p* = .039); and (vi) emotion recognition (GERT—Emotion recognition task; *H* = 8.46, *p* = .015). None of the other effects were significant (*p* > .05 for all comparisons) (Figure 2 and SI 4).

#### Memory encoding

Moderate patients scored significantly higher on the RL/RI 16—Immediate recall than severe patients after FDR correction (*z* = −2.43, *p* = .015), but the other two pairwise comparisons were not significant after FDR correction.

#### Long‐term episodic verbal memory

Mild patients scored significantly higher on the RL/RI 16—Sum of three free recalls than severe patients after FDR correction (*z* = −2.95, *p* = .003), but the other two pairwise comparisons were not significant after FDR correction. Mild patients scored significantly higher on the RL/RI 16—Delayed free recall than severe patients after FDR correction (*z* = −3.26, *p* = .001), but the other two pairwise comparisons were not significant after FDR correction (see Figure 2).

#### Mental flexibility

Mild patients performed the TMT B—Time significantly faster than moderate patients did (*z* = −2.70, *p* = .007), but the other two pairwise comparisons were not significant. Mild patients also performed the TMT B/A—Time significantly faster than moderate patients did (*z* = −2.62, *p* = .009), but the other two pairwise comparisons were not significant (see Figure 2).

None of the other comparisons survived FDR correction (i.e., RL/RI 16—Sum of three total recalls; Stroop Interference—Time; TMT B—Perseveration errors; WAIS IV—Puzzle and Matrix; GERT—Emotion recognition task).

For mean scores and standard deviations, as well as Kruskal–Wallis and Mann–Whitney *U* tests and *p* values, see SI 4.

### 3.2 Structural MRI results as a function of disease severity

No substantial structural damage could be observed. The intergroup structural analysis failed to reveal any significant differences between groups on WM lesions using the mean score on the Wahlund scale. Concerning microbleeds, a single patient had two microbleeds, 18 patients had one microbleed, and 25 had no microbleeds. A significantly higher proportion of mild (55%) patients had at least one microbleed, compared with the moderate (18.50%) and severe (12.50%) patients (see SI 5 and 6).

Similarly, VBM analyses of the structural images could not reveal significant differences of both white and grey matter voxel proportion in the whole brain when comparing the groups. Additionally, while tendential differences were observed in a few regions, comparison of the voxel proportions within the brain parcels were not significant (SI 6).

### 3.3 fMRI connectivity results as a function of disease severity

#### Severe versus mild

**FIGURE 3:**
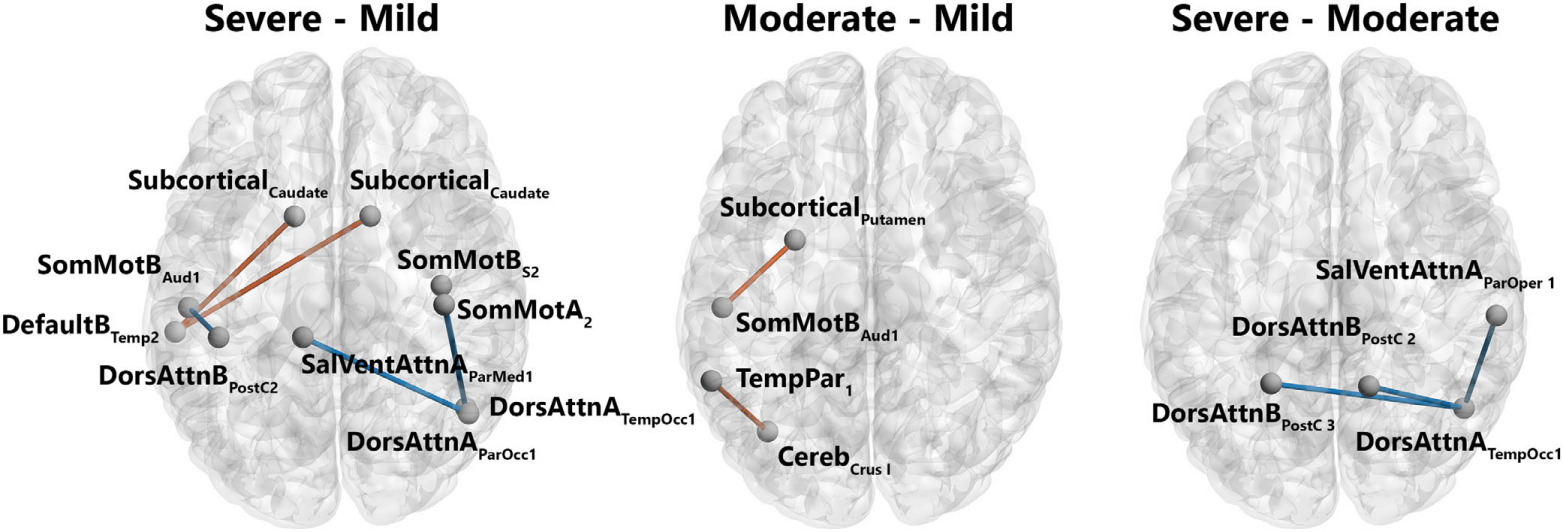
Patterns of significantly different functional connectivity in the intergroup comparison. Differences in functional connectivity between brain structures shown in a network representation on a glass brain when comparing severe versus mild (a), moderate versus mild (b), and severe versus moderate (c). Blue lines indicate a decrease in the connectivity measurement (mean decrease = −0.3), red lines indicate an increase in the connectivity measurement (mean increase = 0.3). Statistical significance was false discovery rate (FDR)‐corrected for multiple comparisons (*p* < .05 FDR). Networks: Cereb: cerebellum; DefaultB: default mode B; DorsAttnA and DorsAttnB: dorsal attention A and B; SalVentAttA: salience ventral attention A; SomMotA and SomMotB: somatosensory motor A and B; TemPar: temporoparietal; Regions: Aud: auditory cortex; ParMed: parietal medial; ParOcc: parietal occipital cortex; ParOper: parietal operculum; PostC: postcentral region; Temp: temporal region; TempOcc: temporo‐occipital region. Figures were created with the BrainNet Viewer (http://www.nitrc.org/projects/bnv/) (Xia et al., 2013)

The connectivity analysis revealed three patterns of hypoconnectivity and one pattern of hyperconnectivity in severe versus mild patients. *Hypoconnectivity*: (i) weaker connectivity between a right temporo‐occipital subregion of the dorsal attention network A (DorsAttn_A_) and two subregions of the somatosensory moto networks A and B (SomMot_A_ and SomMot_B_), respectively; (ii) weaker connectivity the left auditory cortex in the SomMot_B_ network and a postcentral subregion in the dorsal attention network B (DorsAttn_B_); and (iii) weaker connectivity between the right parieto‐occipital subregion in the DorsAttn_A_ network and the left parietal medial subregion in the salience ventral attentional network A (SalVentAttn_A_). *Hyperconnectivity*: higher connectivity was found between a temporal subregion of the left DMN B (Default_B_) and bilateral caudate nucleus in the subcortical network (Figure 3 and SI 7).

#### Moderate versus mild

The connectivity analysis revealed two patterns of hyperconnectivity in moderate versus mild patients: (i) higher connectivity between the left putamen in the subcortical network and the left auditory cortex in the SomMotB and (ii) higher connectivity between the left Crus I in the cerebellum and a subregion of the temporal parietal network (TempPar).

#### Severe versus moderate

The connectivity analysis revealed one patterns of hypoconnectivity in severe versus moderate patients: (i) weaker connectivity between the right temporo‐occipital cortex in the DorsAttn_A_ and the following subregions: bilateral postcentral area in the DorsAttn_B_ network and the right parietal operculum (ParOper) in the SalVentAttn_A_ network.

Anatomical details of the affected regions are shown in SI 7.

### 3.4 Associations between neuropsychological scores and fMRI connectivity as function of disease severity

**FIGURE 4:**
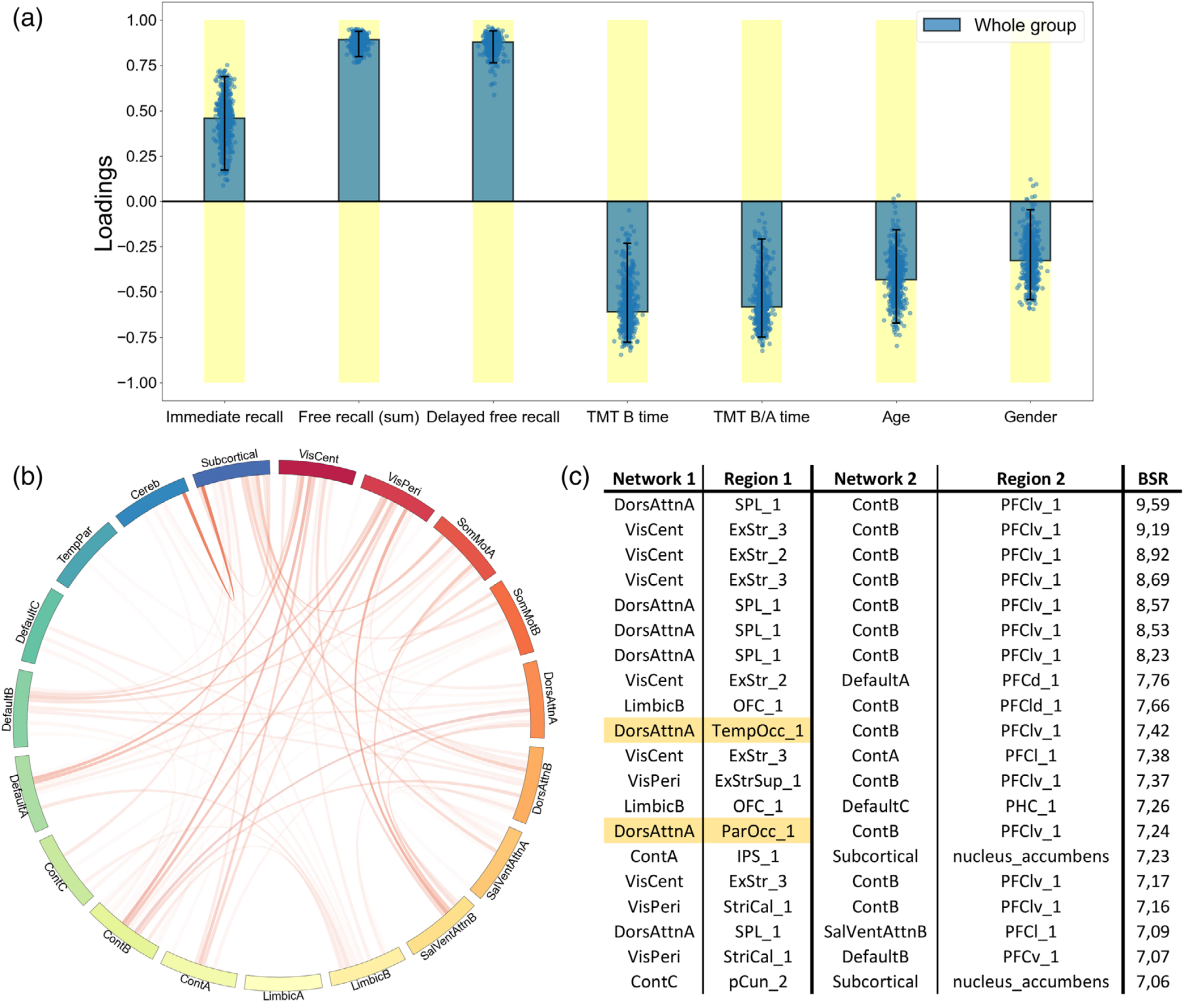
Whole group partial least squares correlation (PLSC), multivariate latent component 1 (*p* = .022, 63.11% covariance). A large positive (or negative) weight indicates a larger contribution of the specific feature to the multivariate correlation pattern. (a) Loadings of the behavioral data. Dots represent samples from the bootstrap procedure, yellow highlights mark weights significantly different from zero, and error bars indicate the 95% confidence interval. (b) Bootstrap sampling ratio (BSR) of the functional connectivity. The network representation illustrates the neuroimaging pattern where red links imply a positive influence of the functional connectivity and darker colors indicate a higher number of connections involved in the pattern. (c) Networks and regions of the top 20 connections with higher impact (BSR) on the latent component. Yellow highlights indicate regions with statistically different connectivities in the group comparison

**FIGURE 5:**
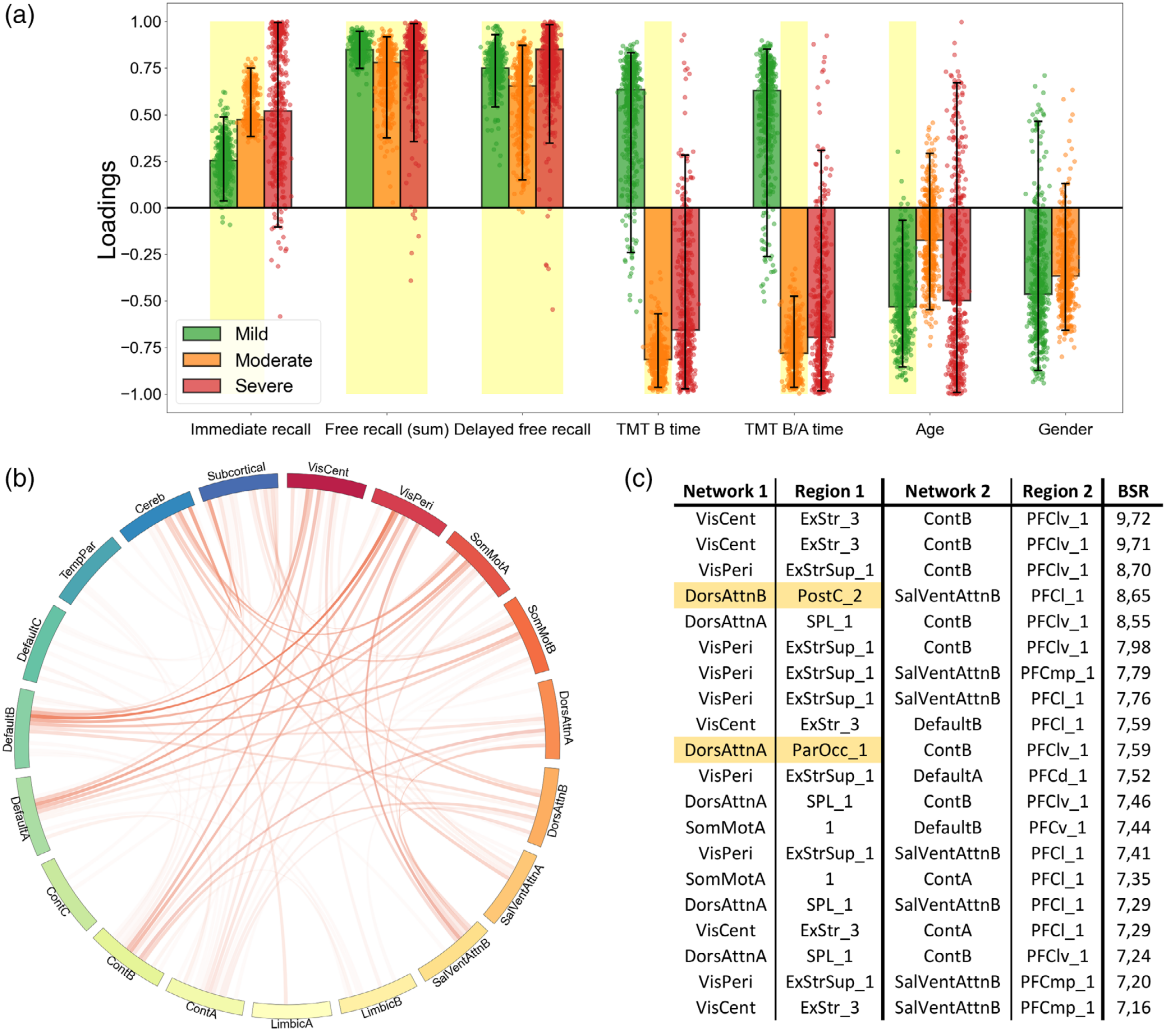
Group‐partial least squares correlation (PLSC), multivariate latent component 1 (*p* = .0019, 35.96% covariance). A large positive (or negative) weight indicates a larger contribution of the specific feature to the multivariate correlation pattern. (a) Loadings of the behavioral data. Dots represent samples from the bootstrap procedure, yellow highlights mark weights significantly different from zero, and error bars indicate the 95% confidence interval. (b) Bootstrap sampling ratio (BSR) of the functional connectivity. The network representation illustrates the neuroimaging pattern where red links imply a positive influence of the functional connectivity and darker colors indicate a higher number of connections involved in the pattern. (c) Network and regions of the top 20 connections with higher impact (BSR) on the latent component. Yellow highlights indicate regions with statistically different connectivities in the group comparison

**FIGURE 6:**
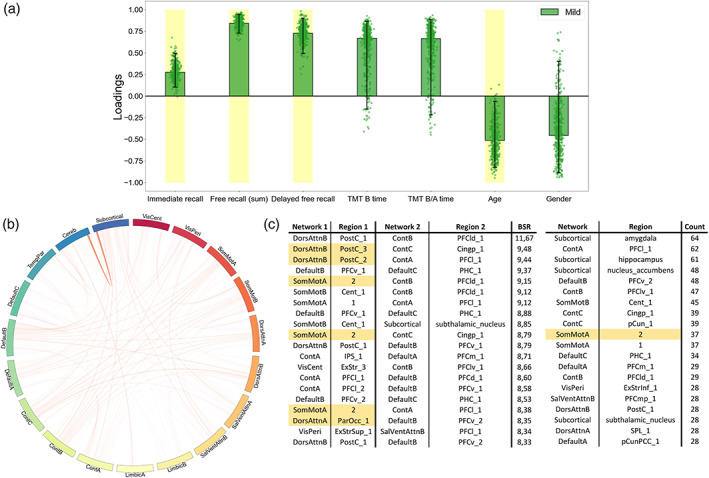
Individual partial least squares correlation (PLSC) on mild patients, multivariate latent component 1 (*p* = .027, 55.99% covariance). A large positive (or negative) weight indicates a larger contribution of the specific feature to the multivariate correlation pattern. (a) Loadings of the behavioral data. Dots represent samples from the bootstrap procedure, yellow highlights mark weights significantly different from zero, and error bars indicate the 95% confidence interval. (b) Bootstrap sampling ratio (BSR) of the functional connectivity. The network representation illustrates the neuroimaging pattern where red links imply a positive influence of the functional connectivity and darker colors indicate a higher number of connections involved in the pattern. (c) Networks and regions of the top 20 connections with higher impact (BSR) on the latent component (left) or with the top 20 highest count in connections from the pattern (right). Yellow highlights indicate regions with statistically different connectivities in the group comparison

The contributions (PLSC loadings) of the different neuropsychological scores and functional connectivity values to the multivariate correlation patterns respective to each PLSC analysis are showed in Figures 4, 5, 6. First, the whole group PLSC analyses extracted one significant component (*p* = .022) explaining 63.11% of the covariance between functional connectivity and neuropsychological data (Figure 4). The extracted component expressed a general trend of correlations between better memory performances (immediate, free, and delayed free recalls) and worse executive performances (TMT B and B/A time) along with younger age and being a woman. Moreover, this behavioral pattern was associated with an increased functional connectivity between the subcortical network and the rest of the brain, notably including the areas from the prefrontal cortex in the executive control network B (Cont_B_), the superior parietal lobule in the DorsAttn_A_ network, the orbitofrontal cortex in the limbic network B (Limbic_B_) and both the central and peripheric visual networks (VisCent and VisPeri).

The group‐PLSC analyses extracted one significant component (*p* = .0019) explaining 35.96% of the covariance between the functional connectivity and neuropsychological data relative to each group (Figure 5). Similar to the whole group component, the multivariate pattern correlates with good performances at the memory tasks with a stable influence of the immediate recall for the mild and moderate patients as well as stable influences of both the free and the delayed free recall for all the groups. However, the poorer executive performances observed in the whole group component were only consistent and reliable throughout the bootstrapping in the moderate group. Although they were not stable, similar trend could be observed in the severe patients while the mild patients displayed better executive performances. The pattern was accurately influenced by a younger age in mild patients. The neuroimaging pattern displayed similar regions with high influence including subregions of the prefrontal cortex in the Cont_B_ and SalVenAttn_B_ network, regions from the SomMot_A_ network and regions from the VisCent and VisPeri networks.

Finally, the individual PLSC analyses on each group yielded a significant component only for the mild group (*p* = .27) explaining 55.99% of the covariance within the data (Figure 6). Again, and mirroring the group‐PLSC results, the behavioral pattern showed stable positive influences of the memory scores along with unstable positive influences of the executive scores and younger age. This behavior pattern was mainly associated with the functional connectivity between the cerebellum and the subcortical networks especially including the amygdala, hippocampus along with both the accumbens and the subthalamic nuclei. Cortical structures such as the somatosensory area, the DorsAttn_B_, Default_B_, and Cont_A_ networks were also involved.

## 4 DISCUSSION

In the present study at 6–9 months post‐SARS‐CoV‐2 infection, behavioral results revealed reduced performance on episodic verbal memory in patients with a severe presentation of COVID‐19, compared with mild and moderate ones, as well as reduced performance on mental flexibility in moderate compared to mild patients. Neuroimaging results confirmed nonstructural alterations of the brain in patients in the long‐term following SARS‐CoV‐2 infection (Guedj et al., 2021; Hosp et al., 2021) but revealed the presence of long‐term patterns of hypo‐ and hyperconnectivity associated with the severity of respiratory symptoms in the acute phase. In detail, when patients with severe disease were compared with mild ones, three patterns of hypoconnectivity were revealed involving subregions of the right SomMotA, bilateral SomMotB, right DorsAttnA, left DorsAttnB and right SalVentAttnA networks. Moreover, one patterns of hyperconnectivity was revealed involving subregions of the subcortical and left DefaultB networks. The comparison between patients with severe disease and moderate ones showed one pattern of hypoconnectivity involving subregions of bilateral DorsAttnB, right DorsAttnA and right SalVentAttnA networks. When moderate patients were compared with mild ones, two patterns of hyperconnectivity were revealed, involving subregions of the left TempPar, left SomMotB, subcortical and cerebellum networks. As for the Douaud et al.'s (2022) study, which compared the structural level of the brain regions in preinfection and postinfection, our results do not highlight structural differences in grey and WM proportions according to the severity of the infection in the acute phase, but this does not exclude that modifications could have occurred in our patients in comparison before the infection. Regarding connectivity results, Voruz, Cionca, et al. (2022), showed cortico‐subcortico‐cerebellar hypoconnectivity patterns in a specific phenotype of patients living with post‐COVID‐19 condition, whereas our results on the severity of infection in the acute phase also showed hypoconnectivity patterns that have not yet been observed to the best of our knowledge. Finally, the multivariate PLSC approach combining behavioral and neuroimaging data revealed significant relationships between cognitive performances and functional brain connectivity as a function of the disease severity. Further analyses showed similar patterns of functional connectivity associated with good performances in the mild patients, while opposite association could be made for the executive functions of the moderate group.

First of all, our results support previous reports of cognitive deficits in the absence of structural brain lesions in COVID‐19 (Khoo et al., 2020; Manganelli et al., 2020; Mohamud et al., 2020; Pilotto et al., 2020). They also suggest that the severity of the initial impairment is a risk factor for the development of long‐term neuropsychological consequences; this could be to a probable post‐ICU/mechanical ventilation effect. The poorer performance for episodic verbal memory displayed by patients with severe disease, compared with mild and moderate patients, partially corroborate the findings of Almeria et al. (2020), who observed reduced neuropsychological performance in ICU patients. Moreover, neuroimaging results revealed patterns of different connectivities in severe patients when compared with both mild and moderate ones including hypoconnectivity in regions from the dorsal attentional networks. An explanatory mechanistic hypothesis for these hypoconnectivity patterns could be alterations of the WM, as suggested by studies in neuropathology (Matschke et al., 2020; Thakur et al., 2021) and in DMI–MRI (Rau et al., 2022). Finally, the PLSC approach revealed stable associations between episodic verbal memory and the dorsal attentional networks suggesting that lower connectivity was coherent with worse memory performances, which is consistent with neuroimaging studies of episodic memory in healthy individuals (see Jeong et al., 2015; Rugg & Vilberg, 2013). Our results also question whether these cognitive effects are solely due to ICU/mechanical ventilation, and perhaps suggest a potential direct or indirect effect of a SARS‐CoV‐2 infection on long‐term neuropsychological consequences. Although the moderate patients were not admitted in ICU and did not undergo mechanical ventilation, they still showed reduced cognitive performance, with reduced performances in mental flexibility in comparison to the mild patients. This corroborates previous behavioral observations by Alemanno et al. (2021), who observed significantly reduced executive scores in patients who received oxygen therapy different than mechanical ventilation but is in contradiction with a recent histopathological study who has observed that changes after COVID‐19 were delimited by those caused by the extracorporeal respiratory assistance treatments (Schwabenland et al., 2021). Moreover, our neuroimaging results revealed three patterns of hyperconnectivity in moderate patients when compared with mild ones, in line with the observations of Zhang et al. (2022) who observed higher interconnectivity pattern of DMN and suggested the involvement of compensatory mechanisms. Finally, and as far as they were concerned, the relationships between behavioral results and brain networks revealed by the PLSC analysis were of opposite sign in the moderate patients when compared with the one of the mild group. Indeed, in the moderate group, higher measures of functional connectivity were associated with poorer scores in mental flexibility. From our point of view, this second pool of results, underlying a pattern specifically displayed by the moderate group, suggests that the neuropsychological reduced performances cannot be solely attributed to a post‐ICU syndrome.

An interesting hypothesis that could encompass the results obtained with the three groups could be a potential alteration of local and global connectivity following a neurological disturbance, in this case, SARS‐CoV‐2 infection. Recent studies in acquired neurological (e.g., cranio‐cerebral trauma), neuroimmunological (e.g., multiple sclerosis) or neurodegenerative (e.g., mild cognitive impairment or Alzheimer's disease) pathologies have highlighted patterns of both higher and lower connectivity (for review, see Hillary et al., 2015). Authors have suggested that hyperconnectivity is a common response following a neurological disruption, but the subsequent depletion of neural resources leads to a rapid decrease in connectivity (Hillary et al., 2015). The presence of compensatory mechanisms inducing patterns of higher connectivity in the short‐term following SARS‐CoV‐2 infection presumably reaches a threshold of cognitive resource availability in the medium term, and eventually leads to a decrease in connectivity and the emergence of hypoconnectivity patterns. This hypothesis is consistent with our results as moderate patients showed greater connectivity than mild patients, while severe patients had lower connectivity in cortical structures, and greater connectivity in subcortical structures (putamen and cerebellum). Severe symptoms in the acute phase may induce a stronger and earlier compensatory response in the cortical networks in the short term and lead to the patterns of hypoconnectivity observed at 6–9 months postinfection, while the subcortical and cerebellar networks may continue to have a compensatory effect. Similarly, patients who had a moderate form in the acute phase may still be in a compensatory mode, thus explaining the increased connectivity compared with mild patients at 6–9 months postinfection. This hypothesis is emphasized by the association between neuropsychological score and functional connectivity in moderate patients, opposite to the one of the mild patients, suggesting that networks are engaged in different processes. Another hypothesis could be a potential alteration of local and global connectivity following a traumatic event in this case, SARS‐CoV‐2 infection that could enhance the effects of SARS‐CoV‐2. Indeed, dysconnectivity is known to be associated with PTSD and consists on hyperactivity and hyperconnectivity of the salience network which has nodes in the insula, dorsal anterior cingulate cortex, and possibly the amygdala.

Considering the behavioral results of our patients combined with neuroimaging results as a function of the severity of SARS‐CoV‐2 during the acute infection, we raise the following considerations. In the case of mental flexibility, which was reduced in the moderate group, studies on healthy subjects have shown the involvement of frontal networks, mainly lateralized to the left hemisphere, in the temporal lobes (left middle and superior temporal gyrus) (Zakzanis et al., 2005), but also the cerebellum (Moll et al., 2002). As discussed above, our neuroimaging results showed increased activation patterns in the temporal cortical networks and in the cerebellum in moderate patients, but no patterns in the frontal lobes. Thus, despite the fact that these networks are involved in the processing of mental flexibility, it is possible that these regions are currently compensating for the other neuropsychological deficits which could therefore induce deficits for mental flexibility by a slowing down the processing speed. According to the literature, such phenomenon could be an important side effect of hyperconnectivity patterns following a neurological disturbance (Hillary et al., 2015). In the case of memory which was significantly reduced in the severe group, neuroimaging studies on healthy subjects have suggested distributed networks of brain regions have been associated with process of encoding, consolidation and retrieval for verbal episodic memory (for review, see Jeong et al., 2015; Rugg & Vilberg, 2013). Interestingly, the majority of studies have demonstrated the involvement of mediotemporal lobe regions (involving hippocampal or parahippocampal structures) in the different processes of verbal episodic memory. However, studies have also shown the involvement of subregions of frontal networks (e.g., dorsolateral prefrontal cortex for the encoding process or medial prefrontal cortex for retrieval). Nevertheless, despite the observed results, the neuropsychological and neurological long‐term effects following SARS‐CoV‐2 are currently unknown, which narrows the scope of interpretation.

Our study has several limitations. By enrolling volunteers, we may have selected the most severe cases, although a significant proportion of our sample did not report any complaints, as confirmed by the very low mean score on the self‐report QPC. This study was only performed on patients who were infected with SARS‐CoV‐2, and these patients had no known clinical history, posing two limitations for generalization of results. Here, we did not include a control group because the aim of the present study was to investigate differences in cognition and brain connectivity as function of the severity of the acute infection. Moreover, with the high rates of infection, it has become more difficult to recruit subjects that have never been infected with SARS‐CoV‐2. Therefore, we cannot exclude that the mild group also exhibits reduced neuropsychological scores in comparison to a control group as has been described in the literature. That said, a recent study by our group did not show a significant accumulation of deficits in the group of mild patients compared to a simulated normative population, while the moderate and severe groups presented a significantly greater accumulation of neuropsychological deficits (Voruz, Allali, et al., 2022; Voruz, Cionca, et al., 2022; Voruz, de Alcântara, et al., 2022). Moreover, our moderate and severe groups are potentially not representative of the population of hospitalized SARS‐CoV‐2 patients because of their lack of comorbidities. It is important to highlight the considerable variance observed in the moderate group, as this could explain the small number of significant differences between groups. The cognitive and psychiatric, as well as functional connectivity (as described above) of the moderate group were extremely heterogeneous, suggesting that some patients presented deficits while others had none, leading to nonsignificant results. The statistical comparison of behavioral data and functional connectivity revealed an imbalance between the groups and the small number of severe participants who underwent MRI may limit the generalization of this group's neuroimaging data. Nevertheless, to the best of our knowledge, studies on functional connectivity in acute and long‐term following SARS‐CoV‐2 infection presented results, on average, from 31 participants with an imbalance in group as function of severity. The acquisition of field maps was not part of the MRI protocol and correction for susceptibility distortion was not performed. Finally, the generalizability of PLS methods has been criticized and, while results stay informative about multivariate correlations within the data, the correlations from PLSC should be validated with techniques such as cross‐validation.

## 5 CONCLUSION

Our study confirms the presence of long‐term neuropsychological effects in patients who had moderate‐to‐severe symptoms in the acute phase of COVID‐19. For the first time, nonstructural alterations of the brain (functional connectivity), associated with neuropsychological performance, were observed in patients, without relevant clinical history at 6–9 months post‐SARS‐COV‐2 infection as function of the severity in the acute phase. Finally, the observed reduced neuropsychological performance 6–9 months postinfection does not solely depend on the severity of the infection in the acute phase.

## CONFLICT OF INTEREST

The authors declare no conflict of interest.

## Supporting information


**APPENDIX S1** Supplementary Information

## Data Availability

At the end of the COVID‐COG project, nonsensitive data will be made available in open access on a dedicated platform.
