# Supplementary material for: Brain functional connectivity alterations associated with neuropsychological performance 6–9 months following SARS‐CoV‐2 infection
Source: Hum Brain Mapp. 2022 Dec 2;44(4):1629–46. doi: 10.1002/hbm.26163 (PMC9878070; doi:10.1002/hbm.26163)
Supplement: Supplementary file 1 — APPENDIX S1 Supplementary Information [file HBM-44-1629-s001.docx]

**Supplementary material**

**Supplementary Information 1. Acquisition parameters for the structural images (T1w MPRAGE, SWI and FLAIR).**

|  | **T1w MPRAGE** | **SWI** | **FLAIR** |
| --- | --- | --- | --- |
| Acquisition time | 4min 36sec | - | - |
| Repetition time | 2300 ns | 27 ms | 5000 ms |
| Echo time | 2.24 ms | 20 ms | 386 ms |
| Acquisition matrix | 256 x 256 | 256 x 256 | 512 x 512 |
| FOV | 186 x 239 x 239 mm | 205 x 205 x 144 mm | 512 x 512 x 192 mm |
| Flip angle | 8 deg | 15 deg | 120 deg |
| Number of Slices | 208 (Sagittal) | 96 (Axial) | 192 (Sagittal) |
| Voxel Size | 0.9 x 0.9375 x 0.9375 mm | 0.8 x 0.8 x 1.5 mm | 1 x 1 x 1 mm |
| Slice thickness | 0.9 mm | 1.5 mm | 1 mm |
| Pixel Bandwidth | - | 120 Hz/pixel | 75 Hz/pixel |
| Inversion Time | - | - | 1800 ms |

**Supplementary Information 2. Acquisition parameters for the functional images (rs-fMRI).**

|  | **rs-fMRI** |
| --- | --- |
| Acquisition time | 7min 59sec |
| Repetition time | 1 s |
| Echo time | 30 ms |
| Acquisition matrix | 88 x 88 |
| FOV | 220 x 220 x 160 mm |
| Flip angle | 50 deg |
| Number of Slices | 64 (Axial) |
| Voxel Size | 2.5 x 2.5 x 2.5 mm |
| Slice thickness | 2.5 mm |

**Supplementary Information 3. MRI motion data as a function of severity in the acute phase and as a function of memory dysfunction awareness**

|  | Severity of acute infection | | | |
| --- | --- | --- | --- | --- |
|  | Mild  (*n* = 20) | Moderate  (*n* = 21) | Severe  (*n* = 9) | K-W |
| Average FD | 0.23 (± 0.1) | 0.29 (± 0.15) | 0.27 (± 0.08) | .418 |
| Excluded Volumes | 7.11 (± 19.94) | 26.6 (± 63.29) | 11.88 (± 21.72) | .496 |

Legend. M-W: FD: framewise displacement; Mann Whitney U test; K-W: Kruskal-Wallis ANOVA test.

**Supplementary Information 4. Mean scores, standard deviations, and intergroup comparisons for cognitive performances among patients who had mild, moderate, or severe COVID-19 at 6‑9 months post-infection**

|  |  | Mild  (*n* = 44)  Mean (± *SD*) | Moderate  *n* = 42  Mean (± *SD*) | Severe  *n* = 24  Mean (± *SD*) | K-W analysis | M-W  Mild  vs.  Moderate  FDR corrected | M-W  Mild  vs.  Severe  FDR corrected | M-W  Moderate vs.  Severe  FDR corrected |
| --- | --- | --- | --- | --- | --- | --- | --- | --- |
| Memory |  |  |  |  |  |  |  |  |
| Verbal episodic memory | Grober & Buschke (RL/RI 16) - Immediate recall | 15.93 (± 0.25) | 15.90 (± 0.30) | 15.33 (± 0.96) | < .001^*^ | .832 | .015^*^ | .024^**^ |
|  | Grober & Buschke (RL/RI 16) - Sum of 3 free recalls | 33.09 (± 5.03) | 30.55 (± 6.53) | 28.25 (± 7.18) | .009^*^ | .053 | .003^**^ | .205 |
|  | Grober & Buschke (RL/RI 16) - Sum of 3 total recalls | 46.66 (± 1.48) | 44.69 (± 3.82) | 44.96 (± 3.42) | .040^*^ | .044^*^ | .029^*^ | .973 |
|  | Grober & Buschke (RL/RI 16) - Delayed free recall | 13.16 (± 2.02) | 12.26 (± 2.56) | 10.96 (± 2.65) | .003^*^ | .140 | .001^**^ | .042^*^ |
|  | Grober & Buschke (RL/RI 16) - Delayed total recall | 15.82 (± 0.45) | 15.36 (± 1.25) | 15.42 (± 1.10) | .057 | - | - | - |
| Visuospatial episodic memory | Rey Figure - Copy time | 158.25 (± 49.63) | 174.05 (± 62.31) | 161.79 (± 79.83) | .281 | - | - | - |
|  | Rey Figure - Score | 34.48 (± 2.21) | 34.19 (± 3.03) | 33.44 (± 3.44) | .579 | - | - | - |
|  | Rey Figure - Immediate recall (3') | 20.41 (± 5.74) | 18.29 (± 6.59) | 17.15 (± 6.08) | .096 | - | - | - |
|  | Rey Figure - Delayed recall (20') | 25.45 (± 5.71) | 22.86 (± 7.27) | 22.52 (± 7.02) | .101 | - | - | - |
| Verbal short-term memory | MEM III -Spans | 9.57 (± 2.02) | 8.62 (± 2.56) | 8.83 (± 2.01) | .079 | - | - | - |
| Visuospatial short-term memory | WAIS IV - Spans | 8.39 (± 2.23) | 7.95 (± 2.15) | 7.46 (± 1.10) | .379 | - | - | - |
| Executive functions |  |  |  |  |  |  |  |  |
| Inhibition | Stroop Interference - Time | 110.86 (± 22.61) | 123.08 (± 31.28) | 131.83 (± 34.27) | .022^*^ | .042^*^ | .016^*^ | .273 |
|  | Stroop Interference - Errors | 0.39 (± 0.78) | 1.03 (± 2.21) | 0.88 (± 1.39) | .150 | - | - | - |
|  | Stroop Interference/Naming - Score | 44.52 (± 14.73) | 52.33 (± 24.75) | 59.46 (± 26.28) | .078 | - | - | - |
| Working memory | MEM III – Verbal working memory | 8.52 (± 1.87) | 8.52 (± 1.97) | 7.83 (± 1.95) | .264 | - | - | - |
|  | WAIS IV – Visuospatial working memory | 8.00 (± 1.83) | 7.64 (± 2.24) | 7.71 (± 1.81) | .679 | - | - | - |
|  | TAP – Working memory item omissions | 2.02 (± 1.98) | 2.53 (± 2.31) | 2.54 (± 2.70) | .649 | - | - | - |
|  | TAP – Working memory false alarms | 3.16 (± 4.55) | 3.60 (± 4.71) | 3.58 (± 3.41) | .778 | - | - | - |
| Mental flexibility | TMT B - Time | 73.66 (± 29.39) | 105.83 (± 75.89) | 102.92 (± 51.03) | .006^*^ | .007^**^ | .008^*^ | .627 |
|  | TMT B - Error | 0.16 (± 0.48) | 0.45 (± 0.99) | 0.46 (± 0.98) | .338 | - | - | - |
|  | TMT B - Perseverations | 0.07 (± 0.25) | 0.67 (± 1.68) | 0.50 (± 0.59) | .002^*^ | .105 | .008^*^ | .351 |
|  | TMT B-A - Score | 42.02 (± 23.65) | 69.24 (± 68.05) | 59.46 (± 26.28) | .007^*^ | .009^**^ | .008^*^ | .558 |
|  | Verbal fluency - Literal (2') | 21.27 (± 5.47) | 21.14 (± 8.00) | 20.79 (± 5.99) | .952 | - | - | - |
|  | Verbal fluency - Categorical (2') | 32.82 (± 8.91) | 31.74 (± 10.89) | 28.29 (± 8.83) | .191 | - | - | - |
| Incompatibility | TAP – Compatibility – False alarms | 0.64 (± 1.16) | 2.88 (± 5.89) | 2.00 (± 6.01) | .413 | - | - | - |
|  | TAP – Incompatibility – False alarms | 1.86 (± 2.31) | 4.70 (± 7.39) | 3.58 (± 6.36) | .535 | - | - | - |
| Attentional functions |  |  |  |  |  |  |  |  |
| Phasic alertness | TAP – Without warning sound – Reaction time | 257.93 (± 46.62) | 278.49 (± 65.38) | 262.96 (± 51.14) | .163 | - | - | - |
|  | TAP – Without warning sound – *SD* of reaction time | 47.09 (± 31.50) | 51.28 (± 28.42) | 49.38 (± 32.10) | .634 | - | - | - |
|  | TAP – With warning sound – Reaction time | 256.27 (± 46.62) | 272.23 (± 53.85) | 256.21 (± 36.80) | .396 | - | - | - |
|  | TAP – With warning sound – *SD* of reaction time | 41.05 (± 17.69) | 48.62 (± 25.39) | 42.33 (± 20.30) | .327 | - | - | - |
| Sustained attention | TAP – Item omissions | 10.50 (± 8.83) | 13.59 (± 11.01) | 11.92 (± 9.20) | .411 | - | - | - |
|  | TAP – False alarms | 5.55 (± 8.05) | 12.41 (± 23.23) | 10.58 (± 13.56) | .085 | - | - | - |
| Divided attention | TAP - Total omissions | 1.64 (± 1.92) | 2.13 (± 2.57) | 2.88 (± 3.80) | .191 | - | - | - |
|  | TAP - Total false alarms | 0.84 (± 1.22) | 1.13 (± 1.68) | 1.75 (± 2.59) | .405 | - | - | - |
| Instrumental functions |  |  |  |  |  |  |  |  |
| Language | BECLA - Semantic image matching | 19.77 (± 0.60) | 19.69 (± 0.64) | 19.63 (± 0.65) | .434 | - | - | - |
|  | BECLA - Semantic word matching | 19.82 (± 0.50) | 19.69 (± 0.64) | 19.63 (± 0.58) | .193 | - | - | - |
|  | BECLA - Object and action image naming | 19.36 (± 0.97) | 19.38 (± 1.17) | 19.21 (± 1.02) | .590 | - | - | - |
|  | BECLA - Word repetition | 15.00 (± 0.00) | 14.93 (± 0.26) | 14.95 (± 0.20) | .210 | - | - | - |
|  | BECLA - Nonword repetition | 9.86 (± 0.51) | 9.79 (± 0.61) | 9.71 (± 0.55) | .228 | - | - | - |
| Ideomotor praxis | Evaluation of ideomotor praxis - Symbolic gestures | 4.93 (± 0.25) | 4.88 (± 0.50) | 4.79 (± 0.41) | .147 | - | - | - |
|  | Evaluation of ideomotor praxis - Action pantomimes | 9.39 (± 0.81) | 9.48 (± 0.92) | 9.38 (± 0.88) | .571 | - | - | - |
|  | Evaluation of ideomotor praxis - Meaningless gestures | 9.39 (± 0.81) | 7.93 (± 0.34) | 7.75 (± 0.74) | .165 | - | - | - |
| Object perception | VOSP - Fragmented letters | 19.41 (± 0.66) | 19.05 (± 2.00) | 19.33 (± 0.76) | .731 | - | - | - |
|  | VOSP - Object decision | 17.61 (± 1.47) | 17.17 (± 2.33) | 17.42 (± 1.98) | .723 | - | - | - |
| Spatial perception | VOSP - Number localization | 9.30 (± 0.95) | 9.05 (± 1.55) | 9.08 (± 1.67) | .929 | - | - | - |
|  | VOSP - Cubic counting | 9.66 (± 0.57) | 9.40 (± 1.74) | 9.67 (± 0.76) | .814 | - | - | - |
| Logical reasoning | WAIS IV - Puzzle | 15.27 (± 5.80) | 12.95 (± 4.52) | 11.92 (± 4.15) | .035^*^ | .060 | .019^*^ | .394 |
|  | WAIS IV - Matrix | 16.75 (± 4.49) | 16.12 (± 5.50) | 13.88 (± 4.35) | .039^*^ | .704 | .010^*^ | .057 |
| Anosognosia | Memory functions | 0.64 (± 0.90) | 0.63 (± 1.15) | 0.35 (± 0.85) | .317 | - | - | - |
| Cognitive complaints | QPC | 3.02 (± 2.18) | 3.15 (± 2.87) | 2.54 (± 2.25) | .645 | - | - | - |
|  | Executive complaints (BRIEF-A) | 106.66 (± 17.20) | 106.53 (± 24.33) | 105.42 (± 19.98) | .849 | - | - | - |
| Emotion recognition | GERT – Emotion recognition task | 25.16 (± 4.98) | 22.10 (± 6.66) | 21.04 (± 6.50) | .015^*^ | .022^*^ | .011^*^ | .535 |

*Note.* FDR: false discovery rate; M-W: Mann‑Whitney *U* test; BECLA: Batterie d’Evaluation Cognitive du Langage; BRIEF-A: Behavior Rating Inventory of Executive Function-Adult Version; GERT: Geneva Emotion Recognition Test; ICU: intensive care unit; MEM-III: Wechsler Memory Scale – Third Edition; QPC: Cognitive Complaints Questionnaire; Rey Figure: Rey-Osterrieth Complex Figure test; RL/RI 16: free/cued recall 16 items; *SD*: standard deviation; TAP: Test for Attentional Performance, Version 2.1; TMT: Trail Making Test; VOSP: Visual Object and Space Perception battery; WAIS-IV: Wechsler Adult Intelligence Scale–Fourth Edition. * *p* < .05. ** *p* < .05 FDR corrected.

**Supplementary Information 5. Results of the visual analysis of MRIs and group comparison between mild, moderate and severe patients.**


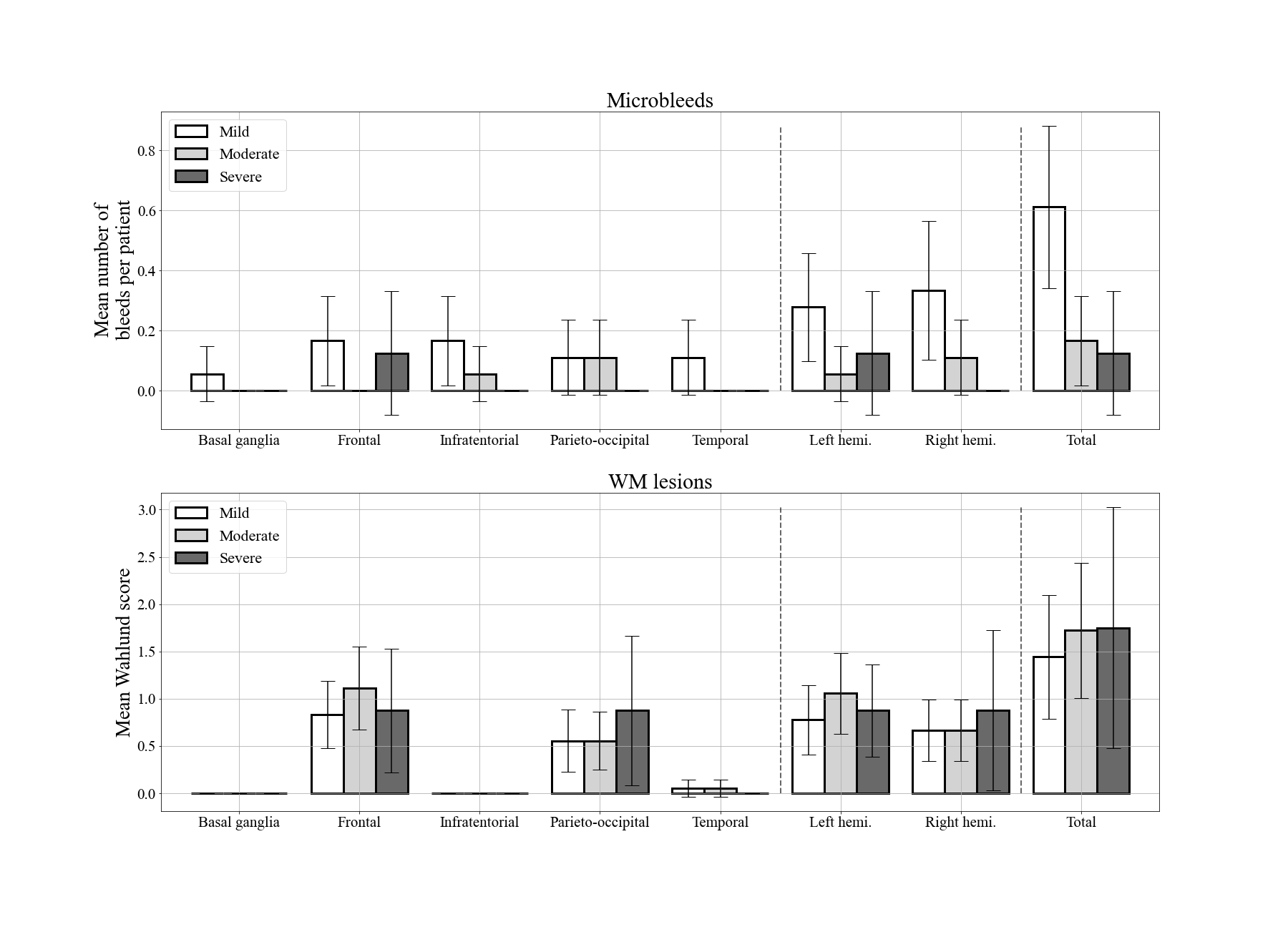


Microbleeds and white matter lesions were quantified respectively from the Susceptibility Weighted Imaging (SWI) and Fluid-attenuated Inversion Recovery (FLAIR) MRI scans**.**

| Whole brain tissue proportions | | | | | | | | | | | | | | | | |
| --- | --- | --- | --- | --- | --- | --- | --- | --- | --- | --- | --- | --- | --- | --- | --- | --- |
|  | **Tissue** | | **Mild, mean** | **Moderate, mean** | | **Severe, mean** | | **Whole group, p** | | **Mild - Moderate, p** | | | **Mild - Severe, p** | | **Moderate - Severe, p** | |
|  | Grey matter | | 0.43640 | 0.42359 | | 0.42014 | | 0.80728 | | 0.46607 | | | 0.47818 | | 0.66096 | |
|  | White matter | | 0.47919 | 0.48187 | | 0.48372 | | 0.97635 | | 0.86421 | | | 0.63334 | | 0.88936 | |
|  |  | |  |  | |  | |  | |  | | |  | |  | |
| Grey matter voxel proportions | | | | | | | | | | | | | | | | |
| Network | **Region** | | **Mild mean** | **Moderate mean** | | **Severe mean** | | **Whole group, p** | | **Mild - Moderate, p** | | | **Mild - Severe, p** | | **Moderate - Severe, p** | |
| DorsAttnB | PostC_3 | | 0.45782 | 0.50928 | | 0.45562 | | 0.04770 | | 0.02375 | | | 0.60736 | | 0.18733 | |
| DorsAttnB | FEF_1 | | 0.52537 | 0.57942 | | 0.55253 | | 0.07191 | | 0.03065 | | | 0.11474 | | 0.57901 | |
| SalVentAttnA | FrMed_1 | | 0.56211 | 0.60249 | | 0.58172 | | 0.20515 | | 0.04774 | | | 0.34413 | | 0.65034 | |
| SalVentAttnB | PFCmp_1 | | 0.69496 | 0.65414 | | 0.64314 | | 0.16425 | | 0.08975 | | | 0.02051 | | 0.96957 | |
| ContC | pCun_1 | | 0.66423 | 0.59525 | | 0.59116 | | 0.11683 | | 0.02985 | | | 0.07592 | | 0.70057 | |
| VisCent | ExStr_2 | | 0.45641 | 0.40671 | | 0.34596 | | 0.13507 | | 0.22232 | | | 0.04002 | | 0.47949 | |
| SomMotA | 3 | | 0.44490 | 0.50444 | | 0.43999 | | 0.07372 | | 0.03491 | | | 0.45950 | | 0.19366 | |
| SalVentAttnA | FrMed_1 | | 0.57845 | 0.61880 | | 0.59364 | | 0.03809 | | 0.02508 | | | 0.60815 | | 0.04645 | |
| Subcortical | globus_  pallidus_  externa | | 0.06101 | 0.08441 | | 0.07371 | | 0.09345 | | 0.04249 | | | 0.53306 | | 0.85609 | |
| Subcortical | amygdala | | 0.81654 | 0.69636 | | 0.73245 | | 0.13341 | | 0.02706 | | | 0.25559 | | 0.24569 | |
|  |  | |  |  | |  | |  | |  | | |  | |  | |
| White matter voxel proportions | | | | | | | | | | | | | | | | |
| Network | **Region** | | **Mild**  **mean** | **Moderate mean** | | **Severe mean** | | **Whole group, p** | | **Mild - Moderate, p** | | | **Mild - Severe, p** | | **Moderate - Severe, p** | |
| DorsAttnB | PostC_3 | | 0.43966 | 0.36147 | | 0.42122 | | 0.03161 | | 0.01464 | | | 0.67991 | | 0.10972 | |
| DorsAttnB | FEF_1 | | 0.42321 | 0.34619 | | 0.37499 | | 0.05656 | | 0.02237 | | | 0.10476 | | 0.40237 | |
| ContC | pCun_1 | | 0.25413 | 0.32661 | | 0.30186 | | 0.09488 | | 0.03056 | | | 0.11696 | | 0.73889 | |
| SalVentAttnA | FrMed_1 | | 0.34007 | 0.29056 | | 0.32855 | | 0.03713 | | 0.02902 | | | 0.98470 | | 0.04214 | |
| ContB | PFCld_1 | | 0.34423 | 0.30385 | | 0.33437 | | 0.08899 | | 0.03810 | | | 0.72924 | | 0.20448 | |
| DefaultA | PFCd_1 | | 0.33167 | 0.28239 | | 0.34209 | | 0.04156 | | 0.03341 | | | 0.97992 | | 0.03773 | |
| Cereb | Vermis_  CrusII | | 0.34001 | 0.41706 | | 0.47450 | | 0.33218 | | 0.30874 | | | 0.02951 | | 0.88677 | |
| Cereb | IX | | 0.42600 | 0.32297 | | 0.36195 | | 0.07760 | | 0.02897 | | | 0.09798 | | 0.65764 | |
| Cereb | IX | | 0.59050 | 0.47749 | | 0.53107 | | 0.09183 | | 0.04232 | | | 0.22373 | | 0.95773 | |
| Cereb | X | | 0.31042 | 0.23433 | | 0.29309 | | 0.09307 | | 0.02265 | | | 0.53844 | | 0.24527 | |
|  | |  | | |  | |  | |  | |  |  | |  | |  |

**Supplementary Information 6. Results of the voxel-based morphometry (VBM) analysis showing the proportion of voxel tissue in the whole brain or within the individual brain parcels.**

**Regions:** ExStr: extra-striate; FEF: frontal eye field; FrMed: frontal medial; ParOcc: parietal occipital cortex; pCun: precuneus; PFC: prefrontal cortex; PostC: postcentral region;

**Supplementary Information 7. Anatomical maps of the affected regions in severe-mild (A), moderate-mild (B) and severe-moderate (C) comparisons.**


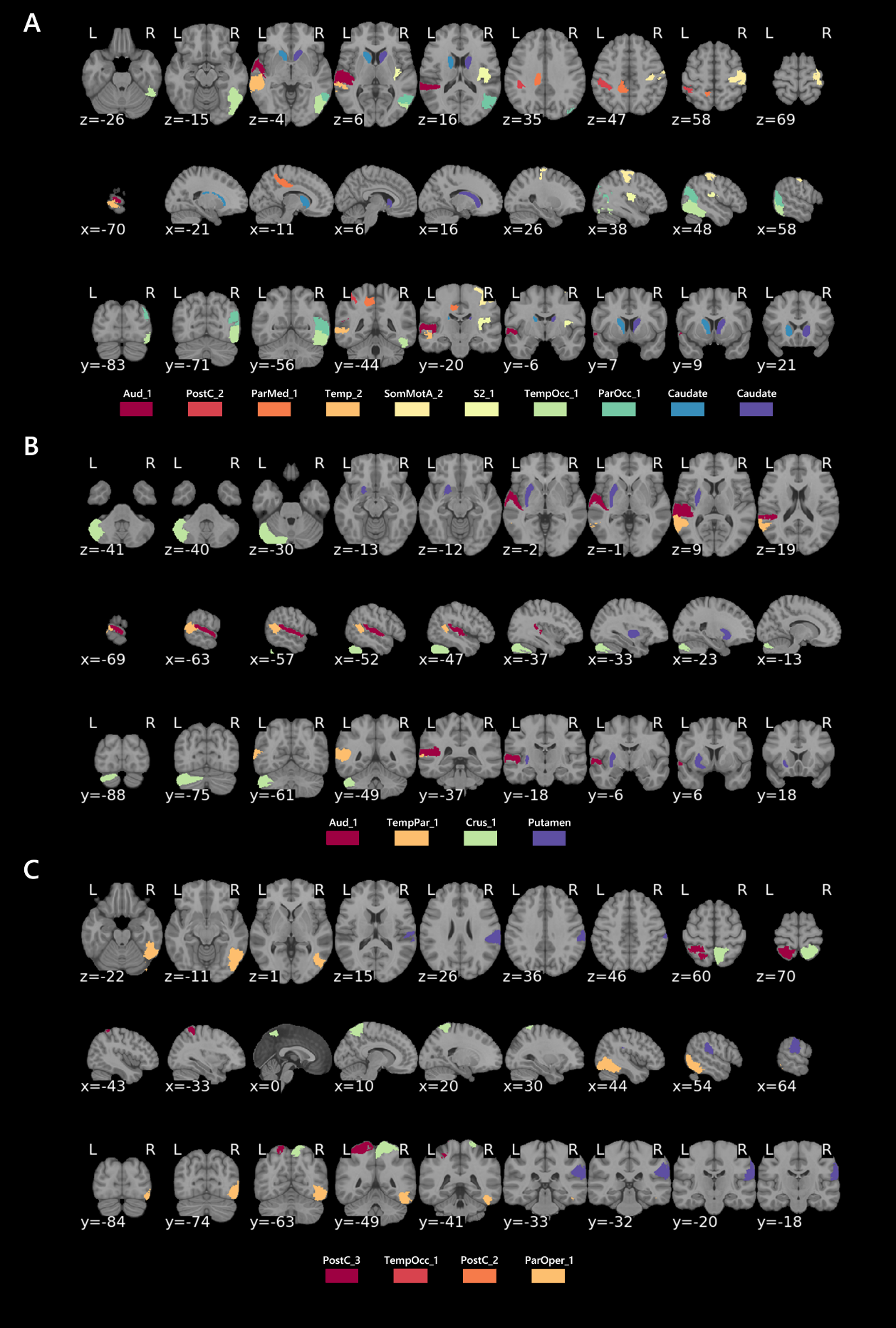


**Regions:** Aud: auditory cortex; ParMed: parietal medial region; ParOcc: parietal occipital cortex; ParOper: parietal operculum; PostC: postcentral region; SomMot: somatosensory motor network; Temp: temporal area; TempOcc: temporo-occipital cortex; TempPar: temporal parietal network.
